# 

**Published:** 1884-10

**Authors:** 


					﻿gj 11
Vol. xx.
October, 1884.
No. 3.
The
iMiiiiiaM
iK/trf £? -J&	lldDR
E □ I T.n r\
ELSOSUMM
				

